# Proteomic analysis of dysregulated pulmonary protein expression and potential pathways in broilers induced by particulate matter exposure in poultry houses

**DOI:** 10.1016/j.psj.2025.105388

**Published:** 2025-06-02

**Authors:** Dan Shen, Kai Wang, Zhendong Guo, Kai Huang, Yansen Li, Chunmei Li

**Affiliations:** a Research Centre for Livestock Environmental Control and Smart Production, College of Animal Science and Technology, Nanjing Agricultural University, Nanjing 210095, China; b Changchun Veterinary Research Institute, Chinese Academy of Agricultural Sciences, State Key Laboratory of Pathogen and Biosecurity, Key Laboratory of Jilin Province for Zoonosis Prevention and Control, 573 Tulip Street, Changchun 130122, Jilin, China

**Keywords:** Broiler, Particulate matter, Lung, Proteomics

## Abstract

Airborne particulate matter (PM) generated by intensive broiler production poses significant risks to poultry respiratory health, yet the molecular mechanisms underlying pulmonary injury remain poorly defined. In this study, PM was collected from a commercial broiler house. Fourteen-day-old AA broilers were exposed to fresh air (control, 0.47 mg·m^-^³), or 4 and 8 mg·m^-^³ total suspended particulates (TSP) for 7 days. iTRAQ-based proteomic profiling identified 4,605 proteins, with 64 differentially expressed (16 upregulated, 48 downregulated) in the high-dose group (8 mg·m^-^³). Enrichment analysis revealed involvement of metabolic pathways, phosphatidylinositol signaling, ATP-binding cassette (ABC) transporters, autophagy, and phagosome-related processes. Key hub proteins—PIK3CD, PIK3C2α, SGK3, and MAP3K20—were highlighted by protein–protein interaction networks and validated by ELISA. Their expression levels correlated significantly with cytokine responses, microbial community shifts, and lung metabolites. RT-qPCR confirmed activation of the MyD88-dependent TLR4/NF-κB and MAPK signaling cascades, along with oxidative stress and apoptotic markers. These findings uncover a PI3K/Akt-centered inflammatory pathway in PM-induced broiler lung injury, offering novel targets for improving poultry respiratory health.

## Introduction

The intensification of poultry production systems has enabled remarkable improvements in meat output efficiency. However, it has concurrently precipitated severe environmental health challenges. Notably, high-density confined poultry houses have become significant sources of airborne pollutants, with particulate matter (PM) emerging as a critical threat to environmental quality and biological health. Extensive evidence indicates that PM accumulation in intensive broiler houses not only contributes to atmospheric pollution—posing risks to neighboring communities—but also directly jeopardizes the health of farmworkers and poultry. Prolonged exposure to elevated PM levels has been linked to respiratory pathologies such as pneumonia, chronic obstructive pulmonary disease, and asthma in humans, with comparable damage observed in avian species.

Among PM fractions, PM_2.5_ (aerodynamic diameter ≤ 2.5 μm) warrants particular attention due to its small particle size (Achilleos et al., 2017) and ability to penetrate deeply into the lower respiratory tract. Approximately 75 % of inhaled PM_2.5_ accumulates in human alveolar regions (Sturm, 2010), where it induces pulmonary inflammation and enters systemic circulation, triggering inflammatory cascades that contribute to both pulmonary (e.g., alveolar epithelial damage) and extrapulmonary disorders (e.g., cardiovascular dysfunction), thereby amplifying its systemic health threats. In birds, the lung rigidly embedded within the vertebral ribs and contains an anastomosing parabronchi network forming interconnected air conduits. In addition, nine air sacs connect with terminal bronchi (Ali, 2020), forming semi-open structures that facilitate deep pentration and retention of inhaled particles. This unique anatomical configuration likely promotes PM_2.5_ deposition at levels even exceeding the 75 % observed in humans, predisposing avian species to heightened susceptibility to airborne pollutants.

PM concentrations in poultry houses have been reported to exceed those in cattle barns and swine facilities, with broiler houses exhibiting higher levels than layer houses (Cambra-Lopez et al., 2010). The total suspended particulate (TSP) concentrations in commercial broiler houses showed an age-dependent increase, with measurements ranging from 0.74 to 11.39 mg·m^-^³. Significantly higher concentrations were observed during winter months (2.10-11.39 mg·m^-^³) compared to summer measurements (0.74-5.25 mg·m^-^³) (Redwine et al., 2002). Current Chinese Agricultural Industry Standards stipulate maximum permissible concentrations of TSP and PM_10_ in poultry houses at 8 mg·m^-^³ and 4 mg·m^-^³, respectively (NY/T 388-1999). However, these regulations notably lack provisions for PM_2.5_ monitoring or threshold limits, despite its predominance in broiler house aerosols and demonstrated pathogenicity. This regulatory gap becomes particularly concerning given that PM_2.5_ concentrations in intensive poultry operations frequently exceed those of ambient air by 10- to 30-fold, creating prolonged exposure scenarios with undefined health consequences for both poultry and occupational workers. Such excessive concentrations correlate with suppressed immune function, reduced body weight gain (Lai et al., 2012), elevated respiratory morbidity, and increased mortality in broilers (Homidan et al., 2003), imposing substantial economic losses. Extensive research has established a definitive association between PM exposure and the pathogenesis of respiratory diseases (Weinmayr et al., 2015; Morales-Suarez-Varela et al., 2017). Nevertheless, effective therapeutic interventions to mitigate the high mortality rates associated with these pathologies remain critically underexplored.

Beyond concentration, the composition of poultry house PM—originating from feed, fecal matter, bedding, and feather debris (Mostafa et al., 2016)—enhances its toxicological potential. These particles often serve as vectors for heavy metals, ions (Shen et al., 2018; 2019), allergens (Li et al., 2014), endotoxins, and pathogenic microorganisms (Dai et al., 2020; Tang et al., 2020). Alveolar macrophages constitute the primary defense against PM invasion. Once deposited in the lung, microbial-associated molecular patterns (MAMPs) on PM_2.5_ surface activate alveolar macrophages via TLR/NF-κB signaling and NLRP3 inflammasome pathways (Feng et al., 2016; He et al., 2017), triggering the release of pro-inflammatory cytokines. These mediators subsequently stimulate alveolar epithelial cells, endothelial cells, and fibroblasts to secrete additional cytokines and adhesion molecules, propagating an inflammatory cascade that culminates in pyroptotic cell death and exacerbated pulmonary tissue damage (Feng et al., 2016). Such inflammatory priming is recognized as a hallmark event preceding clinical manifestations of respiratory diseases. Reported evidence demonstrates that high PM concentrations increase the incidence and mortality of porcine pneumonia and pleuritis while concurrently impairing the phagocytic capacity of alveolar macrophages *in vitro* (Michiels et al., 2015). Although epidemiological and rodent studies have linked PM exposure to respiratory inflammation and epithelial damage, the specific composition of poultry-derived PM_2.5_ and its impact on broiler lung health remain poorly characterized. Our previous studies revealed that PM‑exposed broilers develop hemorrhagic lungs with pale foci; histopathology reveals alveolar septal thickening, macrophage infiltration, and epithelial necrosis, alongside lung microbiota dysbiosis, metabolic perturbations, and histopathological damage, leading to compromised growth performance (Shen et al., 2022, 2023). Abnormal expression of inflammatory and stress‑response proteins suggests involvement of defined signaling pathways, but the molecular regulators orchestrating these lesions are unknown.

Proteins serve as primary effectors of biological responses to environmental stimuli, and proteomic profiling has emerged as a powerful tool for elucidating the molecular mechanisms of toxicant-induced tissue damage (Huang et al., 2014). This approach enables high-throughput identification of differentially expressed proteins (DEPs), functional pathways, and interaction networks (van Ulsen et al., 2009). Particularly in the context of complex environmental stressors like PM_2.5_ (Zhao et al., 2019), proteomics offers critical insights that may not be captured by transcriptomic analyses alone. Recent applications in toxicology have successfully delineated PM-induced protein signatures in mammalian models, but analogous studies in poultry are conspicuously absent. To bridge this gap, we employ isobaric tags for relative and absolute quantitation (iTRAQ)-based quantitative proteomics to map pulmonary protein dynamics in PM_2.5_-exposed broilers, complemented by RT-qPCR and ELISA validation and integrative correlation analyses linking differential expressed proteins with inflammatory markers, microbial shifts, and metabolic perturbations. This multidimensional strategy aims to identify key regulatory proteins mediating PM_2.5_-induced pulmonary inflammation in broilers, delineate associated signaling pathways, and establish mechanistic links between proteomic alterations, microbial changes, and metabolic disruptions. The findings provide novel insights into the pathogenesis of PM-induced lung injury in poultry and offer potential targets for therapeutic intervention and environmental health management in intensive farming systems.

## Materials and methods

### Experimental design

The design of this study, which is a branch of the authors' published trial (Shen et al., 2022), is described briefly as follows: PM was collected from a commercial broiler house located in Yangzhou, Jiangsu Province, China, using a TSP sampler at a height of 1.0 m. The collected PM samples were stored at 4 °C and used in a whole-body exposure experiment to simulate actual rearing conditions. Fourteen-day-old AA broilers were randomly divided into three groups, each consisting of 24 birds. The control group was exposed to ambient air, with an actural TSP concentration of 0.47 mg·m^-3^. In comparison, the medium- and high-exposure groups were subjected to TSP concentrations of 4 mg·m^-3^ and 8 mg·m^-3^, respectively. The TSP concentration of 4 mg·m^-3^ was selected based on actual measurements obtained from commercial broiler houses, representing typical exposure levels under intensive production conditions. The 8 mg·m^-3^ concentration corresponds to the maximum permissible limit for TSP in poultry houses as specified by the Chinese Agricultural Industry Standard (NY/T, 388-1999). Therefore, these two concentrations were chosen to reflect realistic and regulatory-relevant exposure scenarios, allowing for ecotoxicological assessment under field-representative and threshold-level conditions. A 7-day exposure protocol was implemented, where broilers were exposed to PM for 2 hours per day using a dry dispersive injection system (BT901 model, Dandong Baite, China). The PM concentrations in the exposure chambers were monitored in real-time by DustTrak Ⅱ model 8533 aerosol monitors (TSI, USA), and broilers were kept in cages during non-exposure hours. The size-specific PM exposure concentrations across experimental groups and their temporal variations within exposure chambers are systematically documented in Table S1 and Fig. S1, respectively. After exposure, feed consumption and body weight were recorded, and lung tissue was processed for histological analysis and stored for inflammatory cytokine assays, microbiome, metabolomic and proteomic studies. The experimental schedule was as follows: PM exposure was conducted for 2 hours daily over 7 consecutive days. Lung tissue samples were collected 24 h after the final exposure on day 8. Proteins were extracted and quantified within 24 hours, and LC-MS/MS analysis was subsequently performed within 5 days.

This study strictly adhered to the ethical guidelines established by Nanjing Agricultural University's Animal Care and Use Committee and Animal Welfare Committee, as well as the Military Veterinary Research Institute's Laboratory Animal Care and Use protocols.

### Proteomic analysis

Based on the levels of inflammatory factors in lung tissue, four samples from the control group (CON) and the high PM concentration group (8 mg·m^-3^ TSP, PM) were randomly selected for proteomic analysis (Shanghai Meiji Biomedical Technology Co., Ltd.).

**Protein Extraction:** A suitable amount of the broiler lung sample was placed in a shaking tube, and a lysis buffer (8 M urea + 1 % SDS containing protease inhibitors) was added. The tissue was homogenized for 40 s, repeated three times, and then incubated on ice for 30 min, with vortexing every 5 min for 5-10 s. The supernatant was collected after centrifugation at 16,000 g for 30 minutes at 4 °C. Protein concentration was measured using the Pierce BCA protein assay kit (Thermo Scientific, USA), where 20 μL of different concentrations of standard protein solutions were added to the standard wells, and 2 μL of the sample and 18 μL of water were added to the sample wells. Subsequently, 200 μL of BCA working solution was added to each well, mixed, and incubated at 37 °C for 30 min before measuring the OD value at 562 nm of wavelength. The protein concentration was calculated based on the standard curve, and the protein quality was assessed using SDS-PAGE (20 μg loading).

**Enzymatic Digestion, Alkylation, and Labeling:** For enzymatic digestion and alkylation, 100 μg of protein sample was supplemented with lysis buffer to a total volume of 90 μL. A final concentration of 10 mmol/L tris(2-carboxyethyl)phosphine (TCEP) was added as a reducing agent, followed by incubation at 37 °C for 60 min. Then, 40 mmol/L iodoacetamide was added for a 40-minute reaction in the dark at room temperature. Pre-chilled acetone (6:1 v/v) was added, and the mixture was precipitated at −20 °C for 4 h. After centrifugation at 10,000 g for 20 min, the precipitate was dissolved in 50 mmol/L triethylammonium bicarbonate (TEAB) and treated with trypsin (enzyme: protein ratio = 1:50) for overnight digestion.

For iTRAQ labeling and mixing, iTRAQ reagents (ABI, USA) were thawed at room temperature, followed by the addition of acetonitrile and vortex mixing. One vial of iTRAQ reagent was added for every 100 μg of peptide, and the mixture was incubated at room temperature for 2 h. Hydroxylamine was then added for a 15-minute reaction at room temperature, and equal amounts of labeled products were combined into a single tube for vacuum concentration.

**RPLC One-Dimensional Separation:** The peptide samples were reconstituted in UPLC loading buffer and subjected to high pH liquid-phase separation using a reverse-phase C18 column (75 μm × 25 cm). The chromatographic conditions were set with solvent A (2 % acetonitrile, ammonia adjusted to pH 10) and solvent B (80 % acetonitrile, ammonia adjusted to pH 10). The gradient elution program was as follows: 100 % A for 0–2 min; 0–3.8 % B from 2 to 17 min; 3.8–24 % B from 17 to 35 min; 24–30 % A from 35 to 38 min; 30–43 % B from 38 to 39 min; 43–100 % B from 39 to 40 min; and 100 % B for 40–46 min. The UV detection wavelength was set at 214 nm, with a 200 μL/min flow rate, and the total elution time was 66 min. A total of 20 fractions were collected based on peak shape and time, which were then combined into 10 fractions and concentrated via vacuum centrifugation.

**Liquid Chromatography-Tandem Mass Spectrometry:** The peptide segments were reconstituted in mass spectrometry loading buffer and analyzed using nanoliquid chromatography-tandem mass spectrometry (EASY-nLCTM 1200 system and Q Exactive mass spectrometer). After loading, a two-dimensional separation was performed over 120 min using a C18 column. The EASY-nLC liquid chromatography gradient conditions were set with a flow rate of 300 μL/min, using solvent A (2 % acetonitrile + 0.1 % formic acid) and solvent B (80 % acetonitrile + 0.1 % formic acid). The program was as follows: 0–5 % B for 0–1 min; 5–23 % B from 1 to 63 min; 23–48 % B from 63 to 88 min; 48–100 % A from 88 to 89 min; and 100 % B from 89 to 95 min. The mass spectrometry and MS/MS switched automatically, with mass spectrometry resolutions set at 70 K and 35 K, respectively, and a scanning range of m/z 350–1300 (full scan). The top 20 precursor ions were selected for fragmentation with a dynamic exclusion time of 18 s.

**Protein Quality Control and Database Searching:** Raw data from the mass spectrometry were analyzed using Proteome DiscovererTM software (Version 2.2). Statistical analysis and quality control were performed on the high-resolution mass spectrometry results, and the protein sequences of chicken were searched in the NCBI and UniProt databases. The false discovery rate (FDR) for peptide identification was set to FDR ≤ 0.01, and each protein was required to contain at least one specific peptide.

**Data Analysis:** Functional annotation and pathway analysis for the total and differential proteins were performed using Gene Ontology (GO) and KEGG databases. GO and KEGG pathway enrichment analysis for differential proteins was conducted using Goatools software. Fisher's exact test was applied; *P* < 0.05 indicated significant enrichment. A protein interaction network was constructed through network modeling, comparing identified proteins and sequences with the subcellular localization database for subcellular localization prediction. The t-test function in R was used to calculate the significance *p*-values for differences between samples and groups, where significantly DEPs were required to meet the criteria of *P* < 0.05 and a fold change (FC) > 1.2 (significantly upregulated) or *P* < 0.05 and FC < 0.83 (significantly downregulated).

### Key differential proteins assays

Four key differential proteins were detected in lung tissue homogenate supernatant, including phosphatidylinositol-4-phosphate 3-kinase catalytic (PIK3C) subunit type 2 alpha (PIK3C2α), PIK3C subunit type δ (PIK3CD), serum/glucocorticoid regulated kinase family member 3 (SGK3) and mitogen-activated protein kinase kinase kinase 20 (MAP3K20). Lung tissue was homogenized and centrifuged at 3,000 r·min^-1^ for 20 min at 4 °C, and the supernatant was used for analysis. All tests were carried out following the manufacturer's instructions for the respective chicken ELISA kits (Nanjing Jiancheng, China), which employ a competitive assay method. Briefly, 50 μL of standards with varying concentrations and test samples were added to the respective wells. Biotin-labeled antigens were then added, followed by incubation at 37 °C for 30 minutes. After washing 5 times, streptavidin–horseradish peroxidase (HRP) was added and incubated at 37 °C for another 30 min. Plates were washed again (5 times), and substrates A and B were added for color development at 37 °C for 10 minutes. After adding the stop solution, the optical density (OD) at 450 nm was measured within 10 min using a Spark multimode microplate reader (Tecan, Switzerland). A standard curve was generated, and sample concentrations were calculated based on OD values using the fitted equation.

### RT-PCR assay

Lung total RNA was extracted using Trizol reagent (Invitrogen, CA, USA) and then reverse transcribed into complementary DNA (cDNA) using the Transcriptor First Strand cDNA Synthesis Kit (ABclonal, China), following the provided protocol. After assessing the RNA concentration and purity using the NanoDrop 2000 spectrophotometer (Thermo Fisher Scientific, USA), with an OD260/280 ratio, the cDNA was diluted to 500 ng/µL with double-distilled water (ddH_2_O). Quantitative real-time polymerase chain reaction (qRT-PCR) was conducted using the 2 × Universal SYBR Green Fast qPCR Mix kit (ABclonal, China) and the QuantStudio®5 Real-Time PCR Instrument (ABI, USA). Each qRT-PCR reaction mixture consisted of 20 μL, including 2 μL cDNA, 0.4 μL of each primer (10 μM), 10 μL of 2 × Universal SYBR Green Fast qPCR Mix, and 7.2 μL ddH_2_O. This protocol was utilized to measure the expression of genes associated with TLR4/NF-κB, MAPK, and PI3K signaling pathways, as well as inflammatory factors. Relative gene expression levels were normalized to β-actin and calculated using the 2^−ΔΔCt^ method. The gene primer sequences (Supplementary Table S2) were retrieved from the National Center for Biotechnology Information (NCBI) database and synthesized by Shanghai Sangon Biotech Co., Ltd.

### Statistical analysis

Statistical analysis was performed using SPSS version 25, while graphs were created using GraphPad Prism version 8. Unpaired Student’s t-test was employed to identify statistical differences. The Pearson correlation coefficients were computed to measure the strength of the linear association between variables. Data are presented as mean ± standard error of the mean (SEM). Statistical significance was set at *p* < 0.05, while 0.05 < *p* < 0.1 was considered to indicate a tendency toward significance.

## Results

### Functional annotation and subcellular localization prediction of total proteins

**Fig. 1:**
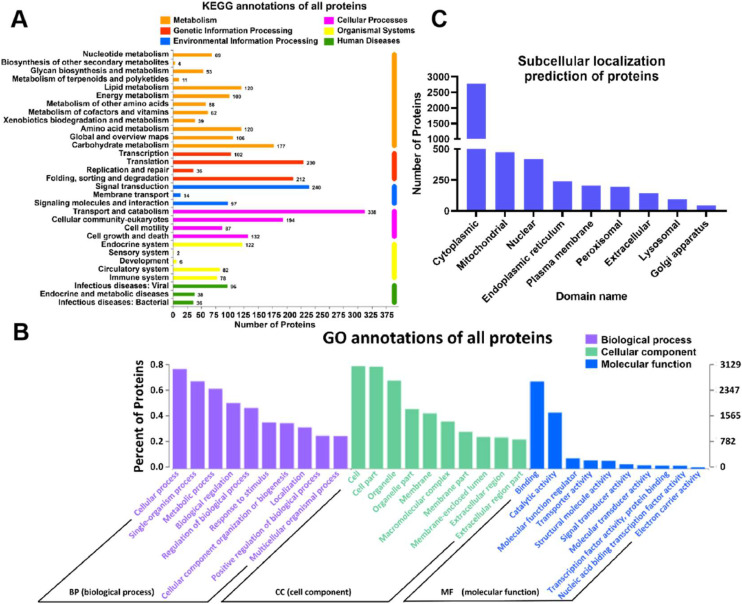
Functional annotation and subcellular localization prediction of total protein in broiler lungs. (A) Function annotations based on Kyoto Encyclopedia of Genes and Genomes (KEGG). (B) Function annotations based on Gene Ontology (GO). (C) Subcellular localization prediction of all proteins. *n* = 4.

A total of 4,605 proteins were identified in the lung tissue of broiler chickens using proteomics. The proteins were subjected to KEGG and GO functional annotations, along with subcellular localization predictions (Fig. 1). As illustrated in Fig. 1A, the KEGG functional annotation revealed that the metabolic pathways were the most enriched. However, when considering the number of proteins, the cellular processes, particularly transport and catabolic functions, accounted for the highest number of proteins at 338. Fig. 1B presents the top ten secondary classifications within the three main categories of GO functions: biological processes, cellular components, and molecular functions. The subcellular localization predictions (Fig. 1C) indicated that most proteins in broiler chicken lung tissue are expressed in the cytoplasm, totaling 2,786 proteins, constituting 60.50 % of the total. Additionally, 476 proteins were identified in the mitochondria and 419 in the nucleus, representing 10.34 % and 9.10 % of the total proteins, respectively.

### Protein expression changes

**Fig. 2:**
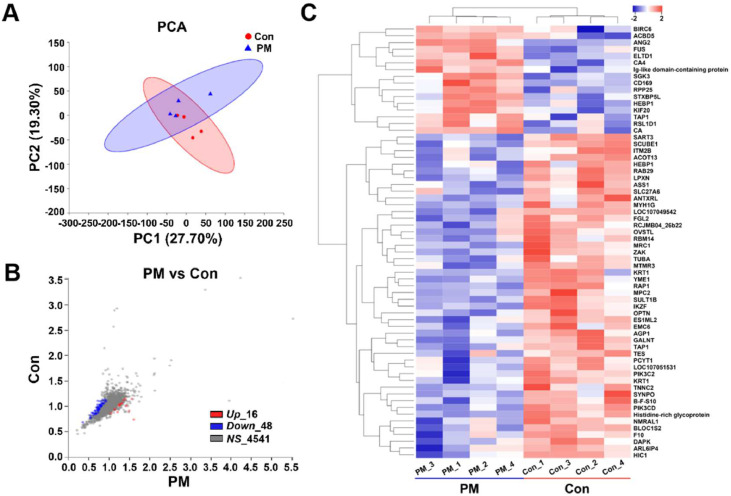
Effects of PM exposure on proteins expression in lungs of broilers. (A) Principal component analysis (PCA) of proteins in lungs. (B) Number of differential proteins in lungs, significant differences were defined as *p* < 0.05 and FC > 1.2 or < 0.83. (C) Clustering of differential protein expression patterns in each sample. *Up*: up-regulated; *Down*: down-regulated; NS: no significant difference. CON: control group; PM: 8 mg/m^3^ TSP group; *n* = 4.

Principal component analysis of proteins from the control and PM exposure groups revealed no significant differences in the overall protein composition (Fig. 2A). The scatter plot of DEPs (Fig. 2B) indicated that, compared to the control group, 16 proteins exhibited significantly increased expression following PM exposure (*p* < 0.05, FC > 1.2), while 48 proteins showed significantly decreased expression (*p* < 0.05, FC < 0.8). The heatmap displayed the relative expression levels of all DEPs (Fig. 2C) and illustrated the clustering of all DEPs and samples. The clustering results demonstrated that the samples within the control and PM exposure groups were more closely related, whereas the inter-group distances were greater.

### Functional annotation of differential proteins

**Fig. 3:**
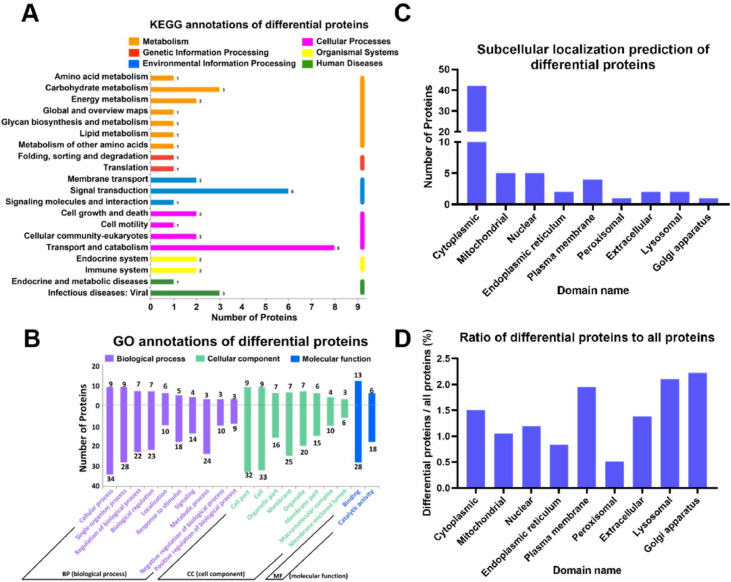
Functional annotation of differential proteins in broiler lungs induced by PM exposure. (A) Function annotations of differential proteins based on KEGG. (B) Function annotations of differential proteins based on GO. (C) Subcellular localization prediction of differential proteins. (D) Percentage of differential proteins subcellular localizations in all proteins. *n* = 4.

Differential proteins in the lung tissue of broiler chickens from the control and PM treatment groups were subjected to KEGG and GO functional annotations, along with subcellular localization predictions (Fig. 3). As shown in Fig. 3A, consistent with the overall protein annotation trends, the KEGG functional annotation indicated that metabolic functions were the most prevalent among the differential proteins. However, when considering the number of proteins, the highest representation was found in cellular processes, particularly in transport and catabolic functions, with 8 proteins. Fig. 3B displays the secondary classifications of GO annotations for all differential proteins and their corresponding primary categories, revealing that most differential proteins were annotated under biological processes, while the least were associated with molecular functions, limited to binding and catalytic activities.

Subcellular localization predictions for the differential proteins induced by PM exposure (Fig. 3C) mirrored those of the total proteins, indicating that the majority, totaling 42 proteins (65.63 %), were localized in the cytoplasm. Additionally, 5 proteins were identified in the mitochondria and nucleus, representing 7.81 % of the total differential proteins. The ratio of the number of differential proteins predicted to localize to the same subcellular compartments compared to the total protein count was calculated (Fig. 3D), revealing that the Golgi apparatus, lysosomes, and plasma membrane contained the highest relative abundance of differential proteins associated with PM exposure.

### Functional enrichment of differential proteins

**Fig. 4:**
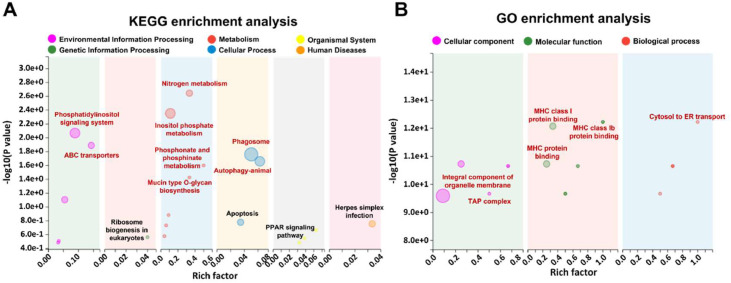
Functional enrichment of differential proteins in broiler lungs induced by PM exposure. (A) Function enrichment of differential proteins based on KEGG. (B) Function enrichment of differential proteins based on GO. *n* = 4.

The differential proteins induced by PM exposure were subjected to KEGG and GO functional enrichment analyses (Fig. 4). As illustrated in Fig. 4A, 8 KEGG pathways were significantly enriched, with the highest number and abundance related to metabolic functions. Notable pathways included nitrogen metabolism, inositol phosphate metabolism, phospholipid and phosphonate metabolism, and mucin-type O-glycan biosynthesis. Additionally, two pathways associated with environmental information processing were significantly enriched: the phosphatidylinositol signaling system and ATP-binding cassette (ABC) transporters. Furthermore, two pathways related to cellular processes were identified: phagosome and autophagy.

Fig. 4B presents the most significantly enriched pathways within the three main categories of GO enrichment. In the category of cellular components, the most prominent pathways included organelle membrane components and tumor abnormal protein (TAP) complexes. For molecular functions, the pathways were primarily associated with major histocompatibility complex (MHC) class I protein binding. In the biological process category, a significant pathway was identified involving protein transport from the cytoplasm to the endoplasmic reticulum (ER).

### Protein interaction network analysis of differential proteins

**Fig. 5:**
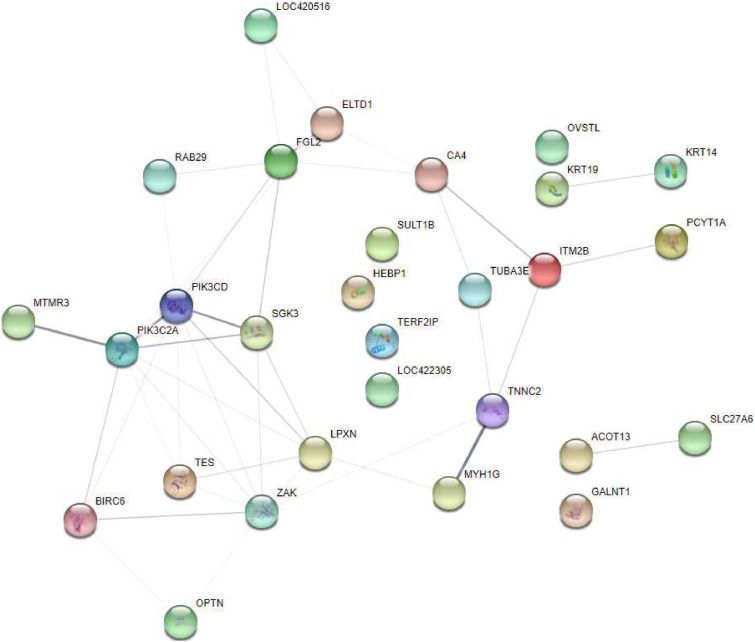
Interactive network analysis of differential proteins in broiler lungs induced by PM exposure.

An interaction network was constructed by linking proteins that exhibited interactions (Fig. 5). The analysis revealed that among the differential proteins, 8 proteins interacted with PIK3CD, 8 proteins interacted with ZAK, which is called MAP3K20 in poultries, 7 proteins interacted with PIK3C2α, 6 proteins interacted with LPXN, 6 proteins interacted with FGL2, and 5 proteins interacted with SGK3.

### Expression of key proteins and mRNAs

**Fig. 6:**
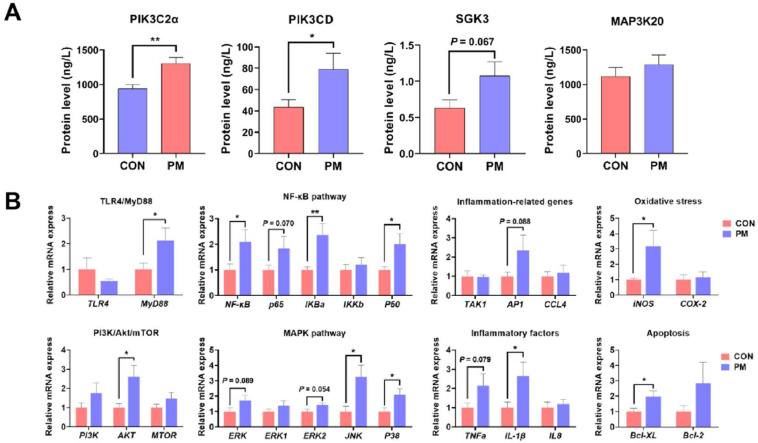
Key differential expressed proteins (A) and genes (B) in broiler lungs induced by PM exposure. *p < 0.05, ^⁎⁎^*p* < 0.01, ^⁎⁎⁎^*p* < 0.001, the numbers indicate *p* < 0.10. *n* = 8.

The analysis of key differential protein expression demonstrated that both isoforms of PIK3C (*PIK3C2α* and *PIK3CD*) exhibited significant upregulation (*p* < 0.05) following particulate matter exposure (Fig. 6A). Additionally, serine/threonine-protein kinase (*SGK3*) showed a tendency toward increased expression (*p* < 0.1).

As shown in Fig. 6B, evaluation of inflammatory pathway-related gene expression revealed markedly elevated mRNA levels of *MyD88, Akt* and NF-κB family components of *NF-κB, IκBα*, and *P50* (*p* < 0.05), with *P65* and *AP1* transcription factors also displaying upward trends (*p* < 0.1). Within the MAPK signaling cascade, stress-responsive kinases *JNK* and *P38* were significantly upregulated, while *ERK* and its isoform *ERK2* exhibited increasing expression tendencies (*p* < 0.1). Pro-inflammatory cytokine profiling indicated a significant rise in *IL-1β* levels (*p* < 0.05), accompanied by a trend of elevated TNFα expression (*p* < 0.1). Furthermore, oxidative stress-associated gene *iNOS* and apoptosis regulator *Bcl-XL* both demonstrated significant increases in expression (*p* < 0.05).

### Correlation analysis of differential proteins with inflammatory cytokines, microbes, and metabolites

**Fig. 7:**
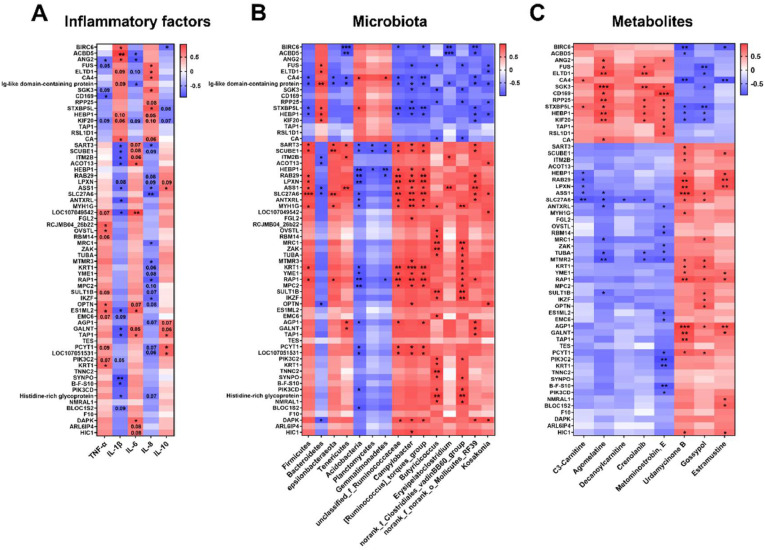
Correlation analysis between differential proteins and inflammation factors level (A), differential microbiota (B), and differential metabolites (C) in lungs of broilers. Pearson Correlations between differential proteins and inflammatory factors level. *p < 0.05, ^⁎⁎^*p* < 0.01, ^⁎⁎⁎^*p* < 0.001, the numbers in rectangles indicate *p* < 0.10.

A Pearson correlation analysis was conducted to examine the relationship between PM-induced differential proteins and inflammatory factors (Fig. 7A). The results indicated a significant correlation between the inflammatory factor TNF-α and 7 differential proteins (*p* < 0.05), with a trending correlation with 9 additional proteins (*p* < 0.10). IL-1β was significantly correlated with 17 differential proteins (*p* < 0.05), and showed a trending correlation with 8 proteins (*p* < 0.10). Similarly, IL-6 had significant correlations with 8 differential proteins (*p* < 0.05) and trending correlations with 9 proteins (*p* < 0.10). IL-8 was significantly correlated with 12 differential proteins (*p* < 0.05) and had a trending correlation with 16 proteins (*p* < 0.10). Finally, IL-10 showed significant correlations with 5 differential proteins (*p* < 0.05) and trending correlations with 5 additional proteins (*p* < 0.10).

Significant correlations were observed between DEPs and several dominant bacterial taxa in lung tissue, including differential phyla and the top 30 genera (Fig. 7B). At the phylum level, *Firmicutes, Bacteroidetes*, and *Acidobacteria* were strongly associated with 12, 11, and 19 DEPs, respectively (*p* < 0.05). At the genus level, *unclassified_f_Ruminococcaceae, Campylobacter, [Ruminococcus]_torques_group, Butyricicoccus, norank_f_Clostridiales_vadinBB60_group*, and *norank_f_norank_o_Mollicutes_RF39* showed notable correlations with DEPs expression. Additionally, correlation analysis between lung DEPs and previously identified metabolomic biomarkers revealed that agomelatine, metominostrobin E, urdamycinone B, and gossypol were significantly associated with 17, 22, 23, and 17 DEPs, respectively (Fig. 7C; *p* < 0.05).

## Discussion

The selected TSP concentrations provide a scientifically grounded framework for evaluating PM-induced respiratory toxicity in broilers under real conditions. By incorporating both field-measured (4 mg·m^-^³) and regulatory threshold (8 mg·m^-^³) exposures, this study offers ecotoxicological insights into how commonly encountered and maximum allowable particulate levels affect pulmonary health, thereby informing environmental safety standards and animal welfare in intensive poultry systems. Although broilers are continuously housed in PM-rich environments, their actual exposure fluctuates throughout the day due to periods of activity and rest. In this study, we employed a 2-hour daily inhalation regimen over 7 consecutive days to simulate these intermittent but intensive exposure phases. This subacute exposure model better reflects real-world patterns of PM inhalation in poultry production systems than constant high-dose models. Furthermore, our preliminary trials confirmed that this design induces reproducible pulmonary inflammation and oxidative stress without causing excessive mortality, ensuring both biological relevance and experimental feasibility. Based on histomorphological alterations, inflammatory responses, and antioxidant activity, both the medium-dose (4 mg·m^-^³ TSP) and high-dose groups (8 mg·m^-^³ TSP) exhibited similar trends, with more pronounced changes observed in the high-dose group. Accordingly, lung samples from the high-dose group were selected for proteomic analysis.

### Functional annotation and subcellular localization prediction of total proteins

The KEGG and GO annotations revealed that metabolic functions predominated across the entire protein dataset, while cellular processes, particularly those involved in transport and catabolic functions, were the most abundant in protein count. This finding aligns with previous proteomic studies on avian lung tissue, where proteins involved in metabolic regulation and cellular maintenance were also prevalent (Vijayakumar et al., 2022). The subcellular localization predictions showed that the majority of proteins were cytoplasmic, followed by mitochondria and the nucleus. These findings confirm that the cytoplasm is the central hub for many cellular activities, including protein synthesis and metabolic pathways. The mitochondrial proteins identified might reflect the high energy demand of lung cells, supporting respiratory functions, while the nuclear proteins suggest ongoing gene regulation and cellular signaling processes. This characterization provides a solid baseline for understanding the molecular composition of broiler chicken lung tissue, which can further be explored in the context of pathological conditions such as exposure to PM.

### Diffferential expressed proteins and their functional annotation

Principal component analysis (PCA) revealed no significant overall differences between the two groups, suggesting that PM exposure did not drastically alter the entire protein composition of lung tissue. However, the upregulation of 16 and downregulation of 48 proteins suggest that PM may induce subtle yet significant alterations in the lung proteome, consistent with findings in other species (Jheng et al., 2021; Yue et al., 2023). The clustering analysis further confirmed these findings, demonstrating clear separation between the control and PM exposure groups. This differential expression likely reflects an adaptive response to PM-induced stress, as the lung tissue attempts to cope with particulate exposure's inflammatory and oxidative effects.

Functional annotation of the DEPs following PM exposure revealed several important trends. The KEGG functional annotation suggests that PM exposure primarily impacts metabolic and transport pathways, potentially altering lung cells' energy and nutrient status. Previous research has demonstrated that exposure to airborne particulate matter can disrupt cellular metabolism by triggering oxidative stress and inflammation, leading to changes in protein synthesis and energy production pathways (Dai et al., 2019). The GO functional analysis further supported these findings, with most differential proteins annotated under biological processes, while fewer were associated with molecular functions. This might indicate that PM exposure primarily affects cellular and tissue-level processes, with fewer alterations at the molecular level, potentially reflecting broader physiological responses to environmental stress.

### Subcellular localization of differential proteins

The high number of cytoplasmic proteins suggests that PM exposure may induce changes in the cytoplasmic components responsible for cellular metabolism, signaling, and stress responses. This finding is consistent with studies on other air pollutants, where cytoplasmic proteins were often implicated in adaptive stress responses (Zeke et al., 2016). Moreover, differential proteins in the Golgi apparatus, lysosomes, and plasma membrane reflect alterations in intracellular trafficking and cellular communication, which are critical in responding to environmental stressors. These findings underscore the dynamic changes that occur in the subcellular compartments of lung cells during PM exposure, with potential implications for the pathogenesis of respiratory diseases.

### Functional enrichment of differential proteins

The enriched pathways identified through functional analysis—nitrogen metabolism, phospholipid metabolism, and mucin-type O-glycan biosynthesis—are essential for maintaining cellular integrity and function. Their enrichment suggests that PM exposure may disrupt normal cellular processes such as energy metabolism, membrane dynamics, and mucosal defense. Notably, the enrichment of pathways related to the phosphatidylinositol signaling system and ABC transporters indicates that PM exposure may affect cellular communication and ion transport, potentially leading to alterations in lung homeostasis. These pathways have been implicated in previous studies of respiratory stress, where changes in signaling and transport mechanisms were associated with lung inflammation and injury (de Groot et al., 2020; Liu et al., 2020). Furthermore, the identification of autophagy, phagosome and the cytosol to ER transport-related pathways suggests that PM exposure may modulate cellular clearance mechanisms, potentially exacerbating inflammation and tissue damage, and this may also be associated with ER stress (Lu et al., 2024; Zhang et al., 2022).

### Protein interaction network analysis of differential proteins

The protein interaction network analysis revealed key interactions between several differential proteins and notable hubs such as PIK3CD, ZAK, and PIK3C2A. These proteins are involved in important cellular processes such as signal transduction, cell survival, and apoptosis. The interaction with PIK3CD, in particular, suggests that PM exposure may activate the phosphoinositide 3-kinase (PI3K) pathway, which is involved in cell survival and inflammation (Garcia-Cuellar et al., 2020). The interaction of proteins with other key players like LPXN, FGL2, and SGK3 further implicates cellular signaling and stress responses in the lung tissue's reaction to PM exposure. Identifying these protein networks provides valuable insight into the molecular mechanisms underlying PM-induced lung injury, highlighting potential therapeutic targets for mitigating the adverse effects of particulate matter.

### Expression of key proteins and mRNAs

Following PM exposure, the significant upregulation of both *PIK3C2α* and *PIK3CD*, along with a trend toward increased *SGK3* expression, suggests that the PI3K/Akt signaling pathway plays a pivotal role in mediating pulmonary responses to environmental insults. PI3K activation is a well-established early event in cellular stress signaling, contributing to inflammation, oxidative stress, and apoptosis (Song et al., 2020). SGK3, a downstream effector of PI3K, has also been implicated in promoting cell survival and ion channel regulation during inflammatory challenges (Liu et al., 2018), indicating a potential compensatory mechanism to counteract PM-induced injury. The analysis of the mRNA expression levels further supports the inflammatory response observed at the protein level, with notable increases in the expression of several key signaling pathways involved in immune response and oxidative stress. Notably, the PI3K/Akt/mTOR pathway, as indicated by the mRNA data for AKT and PI3K, was significantly activated in the PM-exposed group, which aligns with the protein level changes observed for PIK3C2α and PIK3CD. These pathways are central to cellular responses to stress, and their activation is likely a part of the adaptive mechanisms that the lung tissue employs to cope with PM-induced inflammation and oxidative damage (Fu et al., 2023; Ling et al., 2024). Moreover, the oxidative stress markers iNOS and COX-2 were significantly elevated, further supporting the role of oxidative stress in the pathophysiology of PM-induced lung injury.

PM exposure led to marked increases in mRNA levels of *MyD88, Akt*, and key NF-κB family members including *IκBα* and *P50*, with upward trends observed for *P65* and *AP1*. These findings highlight the activation of the MyD88-dependent Toll-like receptor pathway, which is central to initiating innate immune responses and promoting pro-inflammatory signaling through NF-κB and MAPK cascades (Wu et al., 2020). The persistent activation of NF-κB is a known driver of airway inflammation and tissue damage in respiratory pathologies triggered by particulate pollutants (Wang et al., 2022). Similarly, the significant upregulation of *JNK* and *P38* within the MAPK family—both stress-activated kinases—further supports their involvement in mediating cellular responses to oxidative stress and inflammatory cytokine production (Zheng et al., 2022). The observed trend toward *ERK* and *ERK2* upregulation suggests their potential role in tissue repair or proliferation in the later stages of injury response. Moreover, the elevated expression of *IL-1β* and the trend toward increased *TNFα* reinforce the PM-induced inflammatory phenotype, which is consistent with the protein level results we examined (Shen et al., 2022). IL-1β is a key mediator of acute inflammation and is often linked to tissue injury and the amplification of immune responses (Dinarello, 2011), while TNF-α plays a central role in orchestrating NF-κB activation and cell death. The significant induction of iNOS indicates the involvement of nitric oxide in oxidative stress, consistent with prior findings that link iNOS-derived reactive nitrogen species to PM-induced lung toxicity (Prado et al., 2019). The upregulation of *Bcl-XL*, an anti-apoptotic member of the *Bcl-2* family, may reflect a cellular attempt to resist apoptosis and maintain epithelial barrier integrity; however, its prolonged activation could also contribute to pathological remodeling or tumorigenic microenvironments (Geng et al., 2018). These data suggest that PM exposure activates a network of signaling pathways centered on PI3K/Akt and NF-κB/MAPK axes, driving inflammation, oxidative stress, and altered cell survival responses in broiler lungs. These findings provide mechanistic insights into the molecular toxicity of PM and offer potential targets for therapeutic intervention.

This complex interaction between inflammation, oxidative stress, and cellular survival mechanisms highlights the multifaceted nature of lung responses to particulate matter. It underscores the need for further investigation into the precise molecular mechanisms. Clinically, these findings support developing inhalable or feed‑additive modulators targeting the PI3K/Akt and TLR4/NF‑κB axes—such as siRNA‑loaded nanoparticles or plant‑derived PI3K inhibitors—to mitigate PM‑induced inflammation in broiler flocks. Implementing such interventions in poultry production could alleviate the health crisis posed by airborne particulates, improving welfare and reducing economic losses.

### Correlation analysis of differential proteins with inflammatory cytokines, microbes, and metabolites

Lastly, the correlation analysis between differential proteins and inflammatory markers such as TNF-α, IL-1β, IL-6, IL-8, and IL-10 revealed significant associations. These findings suggest that the differential proteins identified in this study likely mediate the inflammatory response to PM exposure, a hallmark of lung injury. Previous studies have demonstrated that PM releases pro-inflammatory cytokines, activating signaling pathways that exacerbate lung damage (Li et al., 2021). The strong correlation with IL-10, an anti-inflammatory cytokine, suggests that some differential proteins may also play a role in modulating inflammation and tissue repair resolution. These correlations underscore the complex interplay between protein expression and inflammatory responses in the lung tissue during PM exposure.

Although sterile lung injury exists in experimental or certain controlled clinical settings, broilers in commercial poultry houses are continuously exposed to complex bioaerosols, including PM carrying bacteria, fungi, and endotoxins (Dai et al., 2020; Tang et al., 2020). In this real-world context, lung injury is rarely sterile. Numerous studies have confirmed that the onset and progression of pulmonary diseases are closely linked to the structure of the lung microbiota (Dickson et al., 2020; O’Dwyer et al., 2019; Budden et al., 2019). Environmental PM not only introduces viable microorganisms but also disrupts pulmonary microbial homeostasis, exacerbating inflammation and tissue damage. Therefore, we considered it biologically meaningful to analyze the correlations between DEPs and microbiota alterations. This integrative approach better reflects the multifactorial nature of PM-induced lung injury and may help uncover potential microbe–host interaction mechanisms that are often overlooked in sterile models. Moreover, the identified correlations between DEPs and metabolomic biomarkers further support the integrative nature of host responses, where microbiota-derived metabolites or host metabolic adjustments may intersect with proteomic regulation. Therefore, the incorporation of microbiome–proteome and metabolome-proteome correlation analysis provides a more comprehensive view of the underlying mechanisms and helps identify multi-level biomarkers for PM-induced lung injury.

## Conclusions

This study provides a comprehensive proteomic analysis of broiler chicken lung tissue following particulate matter exposure, revealing significant changes in protein expression, functional annotations, and protein networks. The findings highlight the impact of PM exposure on metabolic, transport, and catabolic processes and its potential to disrupt cellular homeostasis through altered signaling and inflammatory pathways. Identifying key proteins and pathways involved in these responses provides valuable insights into the molecular mechanisms of PM-induced lung injury and suggests potential targets for therapeutic intervention. Future studies should further elucidate the specific roles of the differential proteins identified in this study, particularly in inflammatory diseases and environmental stressors.

## Declaration of competing interest

The authors declare that they have no known competing financial interests or personal relationships that could have appeared to influence the work reported in this paper.

## Appendix Supplementary materials

Image, application 1
