# Supplementary material for: Proteomic analysis of dysregulated pulmonary protein expression and potential pathways in broilers induced by particulate matter exposure in poultry houses
Source: Poult Sci. 2025 Jun 2;104(9):105388. doi: 10.1016/j.psj.2025.105388 (PMC12173120; doi:10.1016/j.psj.2025.105388)
Supplement: Supplementary file 1 [file mmc1.docx]

Table S1 Actual exposure concentrations of particulate matter (mg·m^-3^) of different sizes in collected broiler house and each group of experiment. (Shen et al., 2022)

| Item | Broiler house | Experiment chambers | | |
| --- | --- | --- | --- | --- |
|  |  | Control | 4 mg·m^-3^ | 8 mg·m^-3^ |
| PM_1_ | 0.571 ± 0.017 | 0.171 ± 0.026 | 0.924 ± 0.200 | 1.442 ± 0.111 |
| PM_2.5_ | 0.713 ± 0.021 | 0.173 ± 0.025 | 1.055 ± 0.199 | 1.760 ± 0.103 |
| PM_4_ | 0.991 ± 0.029 | 0.176 ± 0.025 | 1.219 ± 0.199 | 2.203 ± 0.101 |
| PM_10_ | 2.499 ± 0.077 | 0.228 ± 0.029 | 2.232 ± 0.202 | 4.599 ± 0.139 |
| TSP | 4.033 ± 0.129 | 0.465 ± 0.094 | 4.172 ± 0.087 | 8.297 ± 0.182 |

Table S2 Sequences and parameters of gene primers used for quantitative real-time PCR.

| Gene | Accession No. | Primer sequences |  |
| --- | --- | --- | --- |
| *TLR4* | NM_001030693.1 | F: 5’ AGGCACCTGAGCTTTTCCTC 3’ | |
|  |  | R: 5’ TACCAACGTGAGGTTGAGCC 3’ | |
| *MyD88* | NM_001030962.5 | F: 5’ TAGACAGCAGCGTGCCAAAG 3’ | |
|  |  | R: 5’ CTGTTCCATGCCCATACGGA 3’ | |
| *NF-κB* | XM_046927265.1 | F: 5’ CAGTCAACGCAGGACCTAAA 3’ | |
|  |  | R: 5’ GTTAGCTGTCTGTCTCCACATC 3’ | |
| *P65* | NM_205129.1 | F: 5’ CACGTTTGGTGCAGTGTCAG 3’ | |
|  |  | R: 5’ ATTGACCTTCATGCCCCTCC 3’ | |
| *IkBα* | NM_001001472.2 | F: 5’ CCACTTGGCGATCATTCACG 3’ | |
|  |  | R: 5’ TCTGGCTGAGGTTGTTCTGG 3’ | |
| *IKKβ* | NM_001031397.1 | F: 5’ GTGGAACTTTGTGGTCGGGA 3’ | |
|  |  | R: 5’ TCCTTGTTTACGACCCAGCG 3’ | |
| *P50* | NM_205134.1 | F: 5’ ATCTAGCTCACAAAGGCAGATATAC 3’ | |
|  |  | R: 5’ GAAGGAGGTCTCTACGCCG 3’ | |
| *ERK* | NM-204105.1 | F: 5’ CAACCTCTCCTACATCGGCG 3’ | |
|  |  | R: 5’ TGGCAGTACGTCTGATGCTC 3’ | |
| *ERK1* | NM_204150.1 | F: 5’ CAATGCTGACCCCAAAGCAC 3’ | |
|  |  | R: 5’ GTGCTTCAGCTACAGGCTCA 3’ | |
| *ERK2* | XM_015275131.2 | F: 5’ TGGATGACTTGCCGAAGGAA 3’ | |
|  |  | R: 5’ GAGGCCACAAGACATGACCA 3’ | |
| *JNK* | NO.395983 | F: 5’ TTGGACTGGCAAGAACAGCG 3’ | |
|  |  | R: 5’ AAGGATAACCTCTGGCGCTC 3’ | |
| *P38* | NO.421183 | F: 5’ GGATATGTGGCTACCCGGTG 3’ | |
|  |  | R: 5’TCCAACGAGTCTCAAAATGAGC 3’ | |
| *PI3K* | XM_052686535.1 | F: 5’ CGGATGTTGCCTTACGGTTGT 3’ | |
|  |  | R: 5’ GTTCTTGTCCTTGAGCCACTGAT 3’ | |
| *Akt* | XM_069009912.1 | F: 5’ AAAACAGAGCGACCAAAGCC 3’ | |
|  |  | R: 5’ TGTCTGCTACAGCCTGGATTG 3’ | |
| *mTOR* | XM_040689168.2 | F: 5’GACGAGATGCTTCTTCCAACC 3’ | |
|  |  | R: 5’CCACAATGCTGCGAATAAAAT 3’ | |
| *TAK1* | XM_015284682.2 | F: 5’ ACCACAGCCTATTCCAAGCC 3’ | |
|  |  | R: 5’ TGAATGGATCTGCGCCTCTG 3’ | |
| *AP-1* | NM_205508.1 | F: 5’ ACATGATGTACCAGGGCTTCG 3’ | |
|  |  | R: 5’ AAATCCTGCGAGTTGACGGG 3’ | |
| *CCL4* | NM_001030360.2 | F: 5’ TGGCAGACTACTACGAGACCA 3’ | |
|  |  | R: 5’ TGTAGTCCTGTACCCAGTCGT 3’ | |
| *TNF-α* | NM_000594.4 | F: 5’ GGGACGGCCTTTACTTCGTA 3’ | |
|  |  | R: 5’ GTCTTTGGGGTACTCCTCGG 3’ | |
| *IL-1β* | NM_204524.1 | F: 5’ TGCCTGCAGAAGAAGCCTCG 3’ | |
|  |  | R: 5’ CTCCGCAGCAGTTTGGTCAT 3’ | |
| *IL-8* | NM_000584.4 | F: 5’ ATCTTCCACCTTCCACATCGG 3’ | |
|  |  | R: 5’ TCCCCTAGCAAGCCCTTTTG 3’ | |
| *iNOS* | NM_204961.1 | F: CAAGAGATGGACAAGGGCCA | |
|  |  | R: CTTTGGGAGCCGGAATCCAT | |
| *COX2* | NM_001167719.1 | F: 5’ CTGCGATTTCGAGCGCATTT 3’ | |
|  |  | R: 5’ CTGTTCTGACATGGGAGGGAG 3’ | |
| *Bcl-XL* | NM_001025304.1 | F: 5’ CTTTCAGCGACCTCACCTCC 3’ | |
|  |  | R: 5’ CCCCCAGTTCACACCATCAT 3’ | |
| *Bcl-2* | NM_205339.2 | F: 5’ CACAGGTGCCTACTGTCGTT 3’ | |
|  |  | R: 5’ CACACTGGGATTCTTCCGCT 3’ | |
| *β-actin* | NM_205518.1 | F: 5’ TGTTACCAACACCCACACCC 3’ | |
|  |  | R: 5’ TCCTGAGTCAAGCGCCAAAA 3’ | |


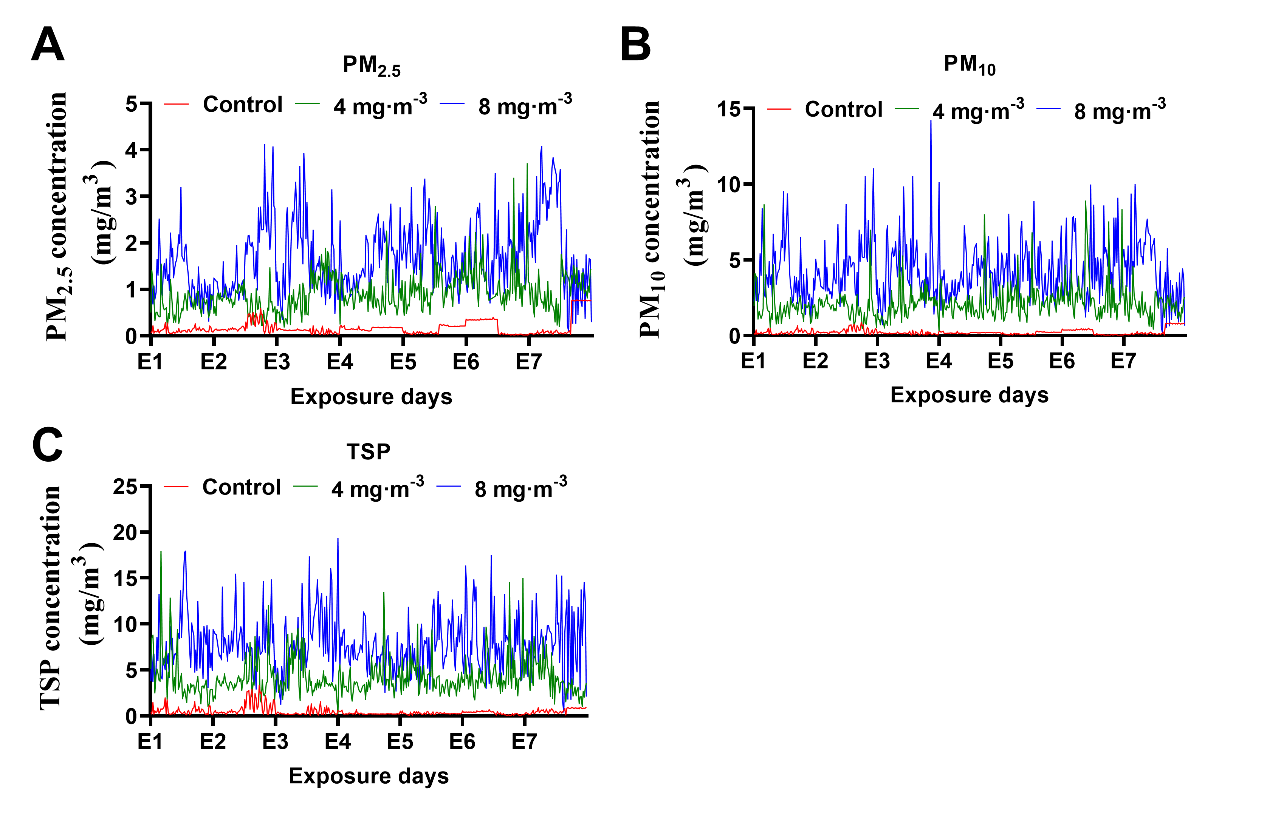


**Fig. S1.** Actual temporal distributions of particulate matter (mg·m^-3^) concentrations in chamber of each group during the experiment. (a) Temporal distribution of PM_2.5_ concentrations in control and exposed groups. (b) Temporal distribution of PM_10_ concentrations in control and exposed groups. (c) Temporal distribution of TSP concentrations in control and exposed groups. E, exposure. (Shen et al., 2022).

**Reference**

Shen, D., Z. Guo, K. Huang, P. Dai, X. Jin, Y. Li, and C. Li. 2022. Inflammation-associated pulmonary microbiome and metabolome changes in broilers exposed to particulate matter in broiler houses. J. Hazard. Mater. 421: 126710.
